# Design, modeling, and manufacturing of high strain composites for space deployable structures

**DOI:** 10.1038/s44172-024-00223-2

**Published:** 2024-06-10

**Authors:** Xiaofei Ma, Ning An, Qiang Cong, Jiang-Bo Bai, Minger Wu, Yan Xu, Jinxiong Zhou, Dayu Zhang, Taotao Zhang, Ruiwen Guo, Huanxiao Li, Yizhe Wang, Xiaotao Zhou, Jialong Zhu, Xin Jin, Yuqing Feng, Di Wu, Tian-Wei Liu, Zhongxi Yan, Tong Wu, Haotian Xi, Qilong Jia

**Affiliations:** 1 China Academy of Space Technology (Xi’ an), Xi’an, 710000 China; 2 School of Aeronautics and Astronautics, Sichuan University, Chengdu, 610065 China; 3 Beijing Institute of Spacecraft System Engineering, Beijing, 100094 China; 4 School of Transportation Science and Engineering, Beihang University, Beijing, 100191 China; 5 College of Civil Engineering, Tongji University, Shanghai, 200092 China; 6 School of Aeronautics and Astronautics, Zhejiang University, Hangzhou, 310027 China; 7 State Key Laboratory for Strength and Vibration of Mechanical Structures, School of Aerospace, Xi’an Jiaotong University, Xi’an, 710049 China

**Keywords:** Aerospace engineering, Mechanical engineering

## Abstract

The demand for larger and lighter mechanisms for next-generation space missions necessitates using deployable structures. High-strain fiber polymer composites show considerable promise for such applications due to their exceptional strength-to-weight ratio, manufacturing versatility, packaging efficiency, and capacity for self-deployment using stored strain energy. However, a significant challenge in using composite deployable structures for space applications arises from the unavoidable extended stowage periods before they are deployed into their operational configuration in orbit. During the stowage period, the polymers within the composites experience material degradation due to their inherent viscoelastic and/or plastic properties, causing stress relaxation and accumulation of plastic strains, thereby reducing the deployment capability and resulting in issues related to recovery accuracy. This paper aims to give a state-of-the-art review of recent advances in the design, modeling, and manufacturing of high-strain composites for deployable structures in space applications, emphasizing the long-term stowage effects. This review is intended to initiate discussion of future research to enable efficient, robust, and accurate design of composite deployable structures that account for the enduring challenges posed by long-term stowage effects.

## Abstract (web summary)

Xiaofei Ma and colleagues provide a review of high-strain composite materials and their use in deployable space structures. The review contributes a broad overview of the field and discusses important design considerations for high-strain composite structures including manufacturing, viscoelasticity, and material selection.

## Introduction

Large and lightweight structures are widely used in spacecraft design, enabling a wide range of applications, including communication antennas, imaging and sensing instruments, radar systems, solar power systems, solar sails, etc^1–3^. Foldable and deployable structures are designed to transition from dramatically different shapes, which are crucial due to the space limitations within the rocket and the need to use that limited space to carry loads and instruments efficiently. In the folded state, they are used to achieve high package efficiency, and in the deployed state, they serve as supporting structures to withstand external loads. High-strain composites (HSCs) are a class of composite material structures engineered to operate at relatively high strains compared to traditional composites. These structures are typically composed of thin plies of fiber-reinforced polymers, designed to achieve high deformations without failure, particularly under bending. The flexibility of HSCs allows them to be folded into a compact volume for transportation and deployed into large-scale structures as needed in orbit, therefore making them well-suited for constructing spacecraft deployable structures. A comprehensive overview of the history of HSCs can be found in a previous review paper organized by Murphey et al.^4^. The use of high-strain structures in spacecraft deployable mechanisms could date back to the 1960s. Early high-strain structures were manufactured from metals such as high strength steel, titanium, aluminum, and beryllium copper alloys. However, metals inherently have high densities, large coefficients of thermal expansion, and lower strain capacities. These properties impose limitations on their use in spaceflight, where missions often demand extreme precision and weight requirements. The limited strain capacity of metals also restricts the achievable geometric and structural architectures. In contrast, fiber-reinforced polymers materials offer advantages such as higher specific stiffness and strength, a low coefficient of thermal expansion, the lightweight properties. Additionally, fiber-reinforced polymers materials are anisotropic and highly tailorable which allows for unique effects upon deformation. Particular HSCs made from fiber-reinforced polymers can be configured to possess two or more stable states which extends the applications compared to metals^5^.

A variety of deployable composite structures have been engineered for both compact folding and autonomous deployment. These structures typically undergo three distinct stages throughout their service life, namely, (1) Folding: Before being launched into space, deployable structures are folded into a compact form. The folding process is usually a slow, quasi-static process where strain energy is stored within the structure as it deforms. Essentially, the structure is carefully manipulated into a shape that can fit within the constraints of the launch vehicle. (2) Stowage: During launch and while being transported into orbit, deployable structures are securely held in their folded state by locking mechanisms. The stowage state can last for an extended period, which may range from months to years, depending on the specific requirements of the mission. (3) Deployment: Once in orbit, these structures are released by unlocking the previously engaged locking mechanisms. HSCs demonstrate versatility in deployment, utilizing both quasi-static methods with the motor roll out and dynamic processes triggered by the rapid release of stored strain energy. This dual deployment capability enhances the adaptability and applicability of HSCs in various engineering contexts.

**Fig. 1:**
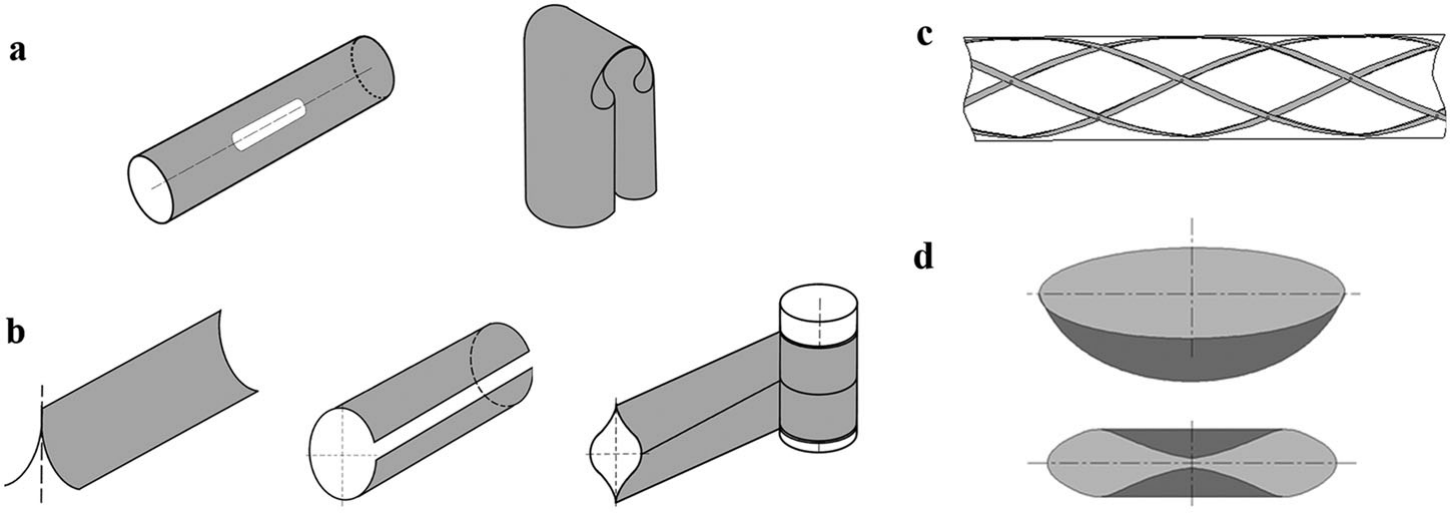
Different configurations of deployable composite structures. **a** Foldable tubes. **b** Collapsible and rollable booms. **c** Elastic extension lattice. **d** Spring-back reflector antenna.

Based on folding methods, deployable composite structures can be categorized into four groups, as shown in Fig. 1: foldable tubes, collapsible and rollable booms, elastic extension lattice, spring-back reflector antenna. The foldable tubes are a typical kind of Deployable Composite Booms (DCBs) created by incorporating cut-out slots in cylindrical tubes, forming tape springs for folding. The foldable tubes are folded by bending at both ends of the structures and thus serve as flexible joints that connect two large panels along their edges. The collapsible and rollable booms represent another category of DCBs designed with specific cross sections, allowing them to be collapsed and rolled up for compact storage and then extended to their full length during deployment. They are usually employed as supporting structures for a variety of large-scale membranes or reflectors in space systems. Indeed, HSC can also be used to manufacture flexible surfaces that can be folded into compact forms along prescribed creases, creating deployable composite structures known as flexible surfaces. Table 1 shows the current research focus and the existing challenges of the deployable composite structures.

**Table 1:** Technical challenges of deployable composite structures

Different configurations	Current focus on research contents	Technical challenges
	Slot optimization	
Foldable tubes	Deployment moment	Mechanical properties after storage
	Failure evaluation	
	Recovery time	
Collapsible and rollable booms	Development stiffness	Mechanical properties after storage
	Manufacturing consistency	Strength at the ends constraint
Elastic extension lattice	Development stiffness	Mechanical properties after storage
	Trust force	
Spring-back reflector antenna	Packaging efficiency	Recovery accuracy after storage

To start with, understanding the folding and deployment behaviors of DCBs is essential for ensuring the functionality of space deployable systems and achieving mission success. Yee et al.^6^ derived the analytical expressions and numerical models to incorporate the orthotropic properties of carbon fiber-reinforced composites for analyzing the moment-rotation behavior of a tape spring constructed from composite laminates. Mallikarachchi et al.^7,8^ conducted extensive numerical and experimental studies to investigate the relationship between quasi-static folding moments and folding angles, as well as the dynamic deployment behaviors for composite tubular foldable hinges. Chen et al.^9–11^ conducted a study on the flattening process of composite thin-walled lenticular tubes (CTLTs) under compression and tension, comparing experimental, numerical, and analytical results. Additionally, Bai et al.^12–17^ studied the stress, deformation, and failure behaviors of CTLTs during folding, utilizing geometrically nonlinear finite element models and an analytical model to characterize the flattening and rolling behaviors of CTLTs. Yang et al.^18^ employed a multi-objective optimization approach to design a C-cross section thin-walled rollable DCB. Their design process involved six consecutive steps in a full simulation, including flattening, end-compacting, releasing, coiling, holding, and deploying around a hub, all based on a nonlinear explicit dynamics analysis.

Furthermore, once DCBs are deployed, they serve as supporting structures that maintain the operational configuration of large-scale space systems. The analysis of strength, stiffness, and stability in deployed DCBs is crucial to ensure their structural integrity under external forces, preventing deformation, instability, or failure. Fernandez et al.^19,20^ investigated various aspects related to folding and deploying behaviors, structural deploying stiffness, shape and ply effects, and fabrication methods for different types of DCBs intended for missions like solar sails and gossamer sail systems. They derived an inextensional analytical model to describe the bending deformation mechanics of collapsible tubular mast (CTM) booms, and explored the impact of varying lamina material, laminate layup, and shell arc geometries between different inner and outer shell segments on the bistability and stiffness properties of CTM. Murphey et al.^21^ analyzed the basic structural mechanics including deployment stiffness, buckling strength, and packaging constraints of triangular rollable and collapsible (TRAC) booms using both closed-form analytical and finite element approaches. Leclerc et al.^22^ performed a study of the nonlinear elastic buckling behavior of TRAC booms under pure bending. Jia et al.^23,24^ explored the nonlinear buckling, post-buckling, and collapse behaviors of CTLTs under pure bending, and revealed the influence of cross-sectional geometry on their stiffness properties and critical buckling loads through a parametric study.

It should be noted that most of the existing studies on the folding and deployment mechanics and stiffness and stability analysis of DCBs have adopted the assumption of linear elastic material properties. However, when DCBs are used in real missions they are often subjected to prescribed loads or enforced displacements for long periods of stowage time. An example of this is a compliant composite flexure in a deployable structure which may be held in a folded, or stowed configuration for many months between assembly and ultimate deployment. Over these timescales, it has been observed that many fiber/resin systems exhibit time-dependent effects which usually, although not always, correspond to degradation of behavior in comparison to predictions made assuming elastic properties or short-term experimental data^25^. This effect caused by the long-term stowage is attributed to the viscoelastic-plastic properties of the polymer matrix^26^. During the long-term stowage period, the DCBs are usually subjected to high-strain deformations, which may result in significant stress relaxation and accumulation of plastic strains. As a result, the stress relaxation dissipates stored strain energy and subsequently diminishes the booms’ deployment capability. The plastic strains can affect the structural integrity of DCBs and lead to issues like reduced stiffness and stability. Moreover, the permanent deformation also affects the shape recovery accuracy of DCBs after deployment, potentially causing deviations of the deployed configurations from the intended configurations. Recognizing that the surface accuracy of space deployable systems, such as antennas and telescopes, significantly impacts their performance, it becomes imperative to incorporate a thorough consideration of the long-term stowage behaviors of DCBs during the design phase. This ensures reliable deployment and the preservation of accurate surface accuracy and overall structural performance.

This paper presents a state-of-the-art review of recent advances in the design, modeling, and manufacturing of HSC for deployable structures in space applications. The key point of this review is firstly to provide a comprehensive and detailed understanding of the shape design and material design of deployable composite structures in order to reduce the folding stress levels while ensure structural stiffness. Then the modeling methods which taken into account the viscoelastic behavior of composite are summarized and the influence of long-term storage is discussed. Finally, the focus is placed on the material selection, manufacturing processes, and functional aspects of deployable composite structure for desired functionalities and shape recovery accuracy.

## Design of deployable composite structures

The design of deployable composite structures in the application of spacecraft needs to strike a balance between flexibility and rigidity to ensure successful folding without material failure and the preservation of structural integrity upon deployment. Beyond this critical balance, there are several aspects that should be considered when designing DCBs to minimize the impact of stowage effects and enhance deployment performance.

### Geometry design of deployable composite structures

**Fig. 2:**
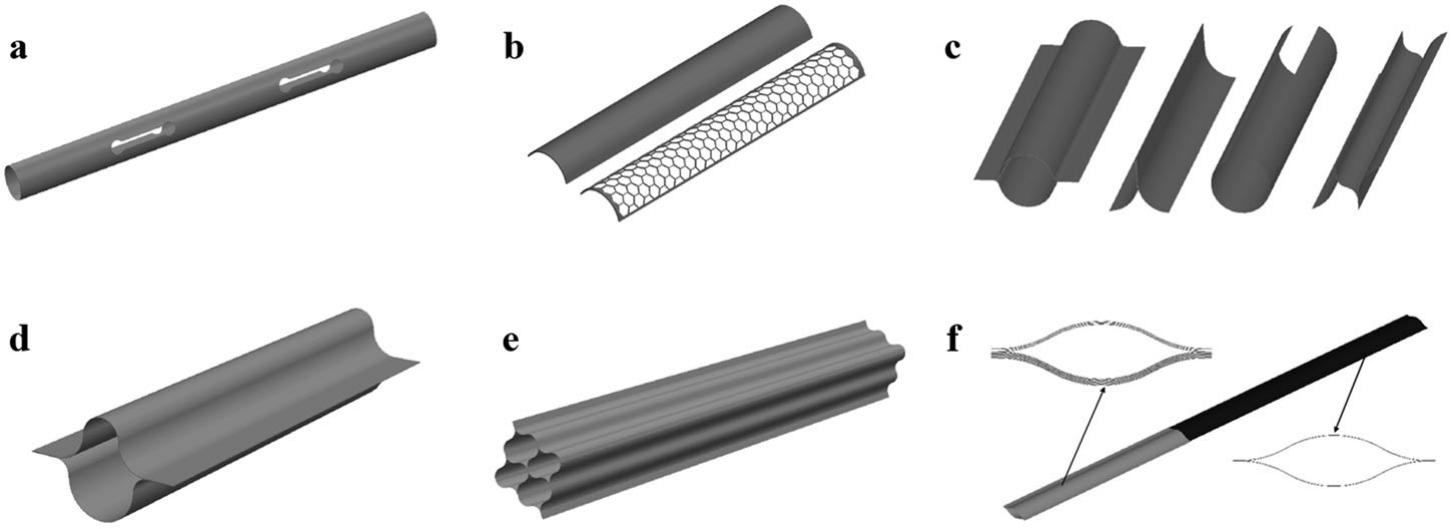
Design of the composite deployable booms. **a** Deployable Composite Boom (DCB) is cut slots near the fold creases^27,28^. **b** The honeycomb topology on the reed structures^31^. **c** The most common cross-sectional shapes of DCB. **d** Two asymmetric omega-shaped shells forms a closed-section^33,34^. **e** Four-cell lenticular combined cross-sections^37^. **f** Eight C-shape combined sections^38^.

In terms of geometry design, DCBs should be shaped to enable as uniform as possible stress distribution at a minimal stress level during folding and stowing, thus minimizing stress relaxation effects. Avoiding stress concentration is also beneficial in reducing the risk of plastic strain accumulation and material failure. As has been discussed in introduction, to facilitate efficient folding without causing material failure, DCBs are engineered by either embedding cut-outs at the folding region or using a collapsible cross-section for roll-up. In the design of cut-outs for foldable DCBs, size, shape, and topology optimization methods were developed to determine the optimal cut-out design for optimal performance. Mallikarachchi et al.^27,28^ proposed a failure criterion for symmetric two-ply plain-weave laminates of carbon fiber-reinforced plastic, and investigated the effect of geometry parameters such as the length, width, and end diameter of the cuts on the failure indices of composite tape-spring hinges, as shown in Fig. 2a. Jin et al.^29^ formulated a cut-out shape optimization for the composite tape-spring hinge (CTSH) in which they aimed to concurrently maximize the maximum strain energy stored during the folding process and the maximum bending moment during deployment while imposing failure constraints. The multi-objective optimization problem was solved by integrating data-driven surrogate modeling and shape optimization. Ferraro et al.^30^ utilized level-set functions to define a variable number of cut-outs in the cut-out topology optimization of foldable joints, which enables damage-free folding while maximizing the stiffness of the structure. Yang et al.^31^ explore the potential of replacing the reed structure with a honeycomb topology, as depicted in Fig. 2b. When compared to traditional spring steel structures, the honeycomb design offers superior mechanical properties per unit mass and can effectively substitute the reed structure. Rakow et al.^32^ introduced the Slit-LockTM boom, designed to provide substantial shear stiffness by incorporating interlocking edge features as the boom unfurls. This relatively new boom technology, equipped with teeth that engage and lock the seam during deployment, enhances stiffness. The Slit-LockTM boom effectively carries bending loads, performing similarly to a closed-section tube, as long as the ends are shear-fixed. Holes can be cut into the entire thin-wall shell.

On the other hand, the rollable DCB is usually composed of thin-walled shells with varying curvatures that are bonded along their edges. The selection of the cross-sectional shape for these DCBs depends upon the technical specifications of the spacecraft. Common shapes include lenticular, triangular, tubular, C-shaped, N-shaped, and more, as illustrated in Fig. 2c. Lee et al.^33,34^ introduced a two-shelled DCB, where two symmetric or asymmetric omega-shaped shells form a closed-section, resulting in high stiffness and dimensional stability, as depicted in Fig. 2d. They developed a two-parameter inextensional analytical model to identify laminates and shell geometries inducing bistability and conducted a parametric analysis to determine optimal configurations that maximize stiffness while retaining bistability. Yang et al.^35,36^ studied triangle-shaped and N-shaped DCBs. Parametric analyses were conducted, and all design variables were found to significantly influence the wrapping peak moment, maximum stress, and deploying fundamental frequency. Notably, the maximum stress showed higher sensitivity to changes in the central radius. Yang et al.^37^ also proposed a new four-cell lenticular honeycomb DCB, as shown in Fig. 2e. Fatigue cracks caused by stress concentration are avoided by setting maximum principal stress to a specific constraint. Cao et al.^38^ introduced a novel combined cross-sectional shape, consisting of eight C-shaped thin-walled shells, as depicted in Fig. 2f. To minimize the maximum stress in the stowage configuration, a gap is maintained between adjacent thin-walled shells. Sharma et al.^39^ investigated slit cross-section overlapped section DCBs and accurately predicted the transition zone length and working stress. To enhance strain energy during storage and the deployment force, a smaller cross-section radius for the DCB is recommended. Furuya et al.^40^ introduced the concept of corrugated closed-section booms to enhance deployment torque, shape restoration performance, and storage efficiency.

Compared to traditional DCBs, the adoption of cut-outs design significantly reduces the folding stress level during folding and stowing, thereby mitigating the risk of stress release effects. However, this design also introduces new challenges such as reducing structural strength in the opening part, and increasing complexity in the manufacturing process. Additionally, it may lead to new failure modes such as local buckling and interlayer shear failure of composite structures. To address these issues, a novel cross-sectional shape design is proposed for these DCBs which enhances their structural stiffness and deployable moment without increasing the folding stress level. Nevertheless, this design poses difficulties in connection, fixing, and manufacturing while considering the surge in total mass of the DCBs. Therefore, it is necessary to multi-objective optimize the topology of the DCBs under various constraints in the future.

### Design of composite material

The selection of composite materials and the configuration of their layup are crucial. The choice of materials, including fiber type, resin, and stacking sequence, not only significantly influences structural flexibility and rigidity but also impacts stress relaxation effects. The DCBs are prepared with carbon fiber/epoxy resin composites conventionally. Augello et al.^41^ analyzed the effect of different materials on the folding of ultrathin tape-spring hinges. The materials include isotropic metallic with hardened steel tape spring and unidirectional T300 graphite fiber/epoxy prepreg. Bowen et al.^42^ presents an extensive study of the minimization of the Brazier moment to enhance the design of orthotropic cylindrical flexible hinges. Su et al.^43^ established the progressive damage model of composite cylindrical thin-walled hinges. To solve the problem of localized folds of bistable composite tubular booms due to local buckling phenomena when the diameter of the coil increased, Fernandez^44^ proposed an improved scheme of a variable angle change over the DCB length. Then, the bending properties of the shell structure are changed at every section, yielding DCBs to naturally coil into a stable spiral as is imposed in reality. Recently, smart composites and high-temperature resistant materials have been applied to the composite deployable booms. An et al.^45^ proposed self-deployable systems by the synergic combination of shape memory alloy-enabled smart soft composites with kirigami/origami reflectors. Roh et al.^46^ proposed a DCB using woven fabric fibers and shape memory composites. The viscoelastic time-dependent unfolding behavior including structural nonlinearity of thin-walled shape memory composite booms are experimentally and numerically investigated. Liu et al.^47^ studied the mechanical properties of shape memory polymer composites-based DCBs. Compared with traditional DCBs, shape memory polymer composites-based DCBs exhibit the advantages of controllability and stability in the deploying process and still work well after folding 30 deformation cycles. DCBs are prepared with epoxy resin composites conventionally, which are prone to undergo glass transition at high temperatures. Zhang et al.^48^ proposed a novel high-temperature resistant carbon fiber/bismaleimide resin composite shell and its bistable characteristics are studied in megathermal environment. The effect of elevated temperatures and different ply angles were investigated on curvatures and snap behaviors of carbon fiber/bismaleimide resin composite bistable shells. The material design method for the DCBs based on carbon fiber/epoxy resin composite is highly refined. Compared to traditional composite materials, some novel composite material introduces exceptional properties to the DCBs, thereby significantly expanding their application potential. However, substantial research efforts are necessary prior to practical application, including precise analysis, optimal design, and experimental validations. The multi-physics coupling analysis of various materials is very important to investigate the mechanical characteristics of the DCBs. Furthermore, a layer optimization model for heterogeneous composite materials also remains an imperative task for future studies.

The influence of layer orientation on creep behavior mainly has two aspects. Firstly, the shear strain of the single layer is related to the orientation angle under specific deformation of the total laminates. Secondly, the stiffness of the laminates has a great correlation with the layer orientation, so the deformation of the laminates is related to the layout under specific external force. Mao et al.^49^ monitored the deployment process of the fiber-reinforced tape springs with laminate layouts of [−45°, 45°], [−45°, 0°, 45°] and [−45°, 0°, 90°, 45°]. The results showed that the four-layer tape springs were self-deployable after more than 6 months of stowage, while the two- and three-layer tape springs lost their self-deployability after a few days of stowage. Singh et al.^50,51^ investigated the creep behavior of the woven glass fiber-reinforced polymer laminates with layouts of [0°, 90°], [15°, −15°], [30°, −30°] and [45°, −45°]. The results showed that as the fiber off-axis angle increased, the specific creep increased. Kang et al.^52^ studied the viscoelastic properties of carbon fiber-reinforced polymer (CFRP) and concluded that the laminates with the layout of [0°, 90°]_4_ showed lower relaxation than [45°, −45°]_4_. The studies mentioned above show that the viscoelastic behavior is related to the layer orientation, and it’s feasible to reduce the influence by changing the laminate layout. However, the relationship between laminate layout and creep behavior has not been revealed yet, which is an important research direction for the future.

## Modeling stowage behavior of HSCs

Multiscale viscoelastic modeling is essential for gaining a deep and comprehensive understanding of the stowage effects in deployable composite structures. By examining the material behavior and structural response at various scales, from the microscale of individual material constituents to the macroscale of the complete structure, engineers can make more informed decisions about material selection, design optimization, and mitigation strategies to address the challenges associated with stowage effects.

### Multiscale modeling of viscoelastic composites

**Fig. 3:**
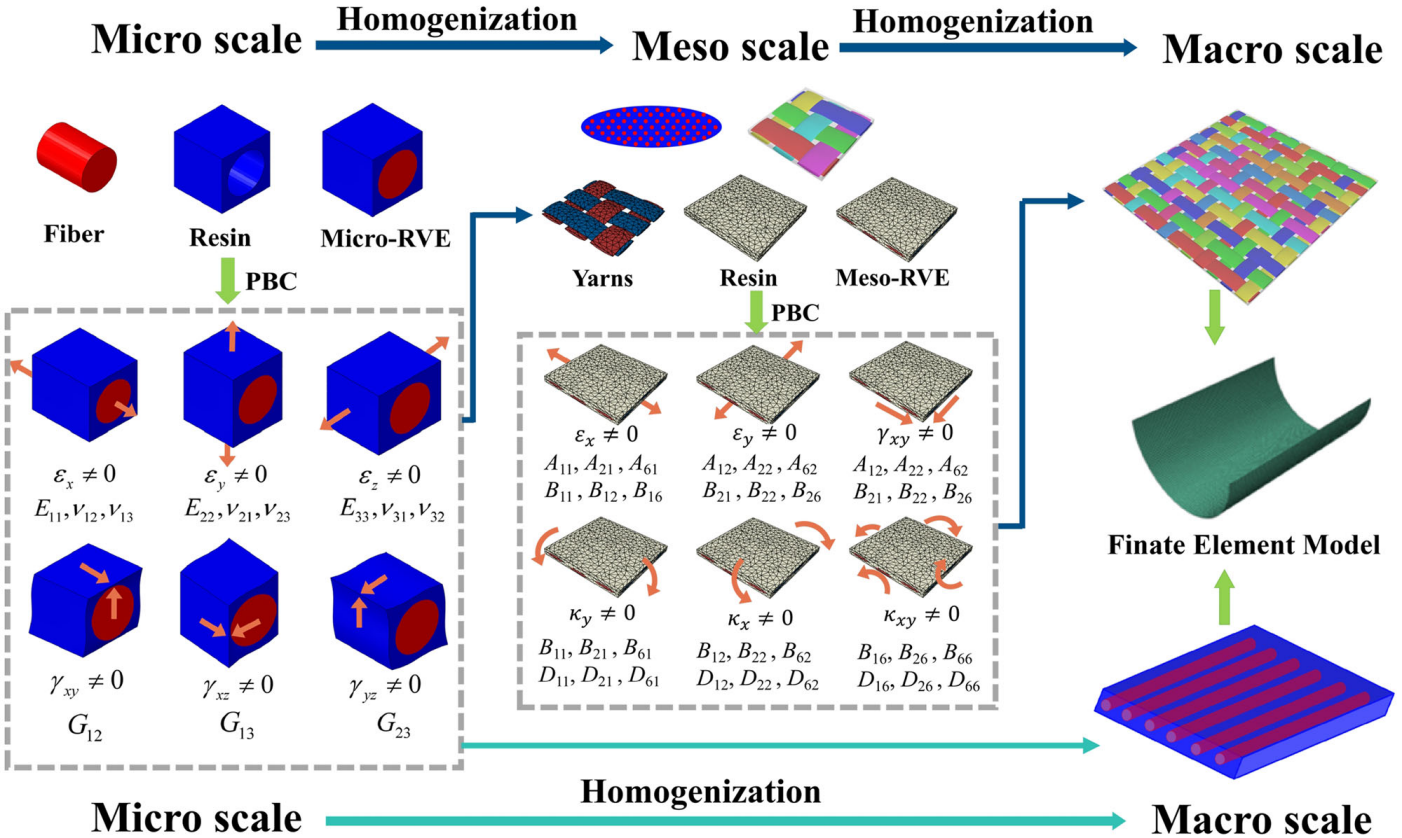
Multiscale modeling framework of homogenization methods for predicting the viscoelastic behavior of composite laminates. The multiscale modeling strategy can be summarized as a two-step process. The first homogenization step involves creating a microscale representative volume element (RVE) that represents the microstructure of the unidirectional composites. The fiber is assumed to be a linear elastic material, while the matrix is assumed as an isotropic, and linear viscoelastic material, which can be described by a generalized Maxwell model using the Prony series. The second homogenization step utilizes the obtained unidirectional composite (yarn) properties to analyze the behavior of the woven composite by constructing a mesoscale RVE.

In terms of the viscoelastic mechanics of composite materials, multiscale modeling techniques are employed to simulate the overall mechanical behavior of laminated composite structures. The modeling strategy can be summarized as a two-step multiscale homogenization method^53–56^, as depicted in Fig. 3. The first homogenization step involves creating a microscale representative volume element (RVE) that represents the microstructure of the unidirectional composites. This step determines the effective material properties of the composite through homogenization of the fiber and matrix properties in the yarn microstructure. The fiber is assumed to be a linear elastic material, either transversely isotropic or isotropic, characterized by its Young’s Modulus and Poisson ratio. The matrix is assumed as an isotropic, and linear viscoelastic material, which can be described by a generalized Maxwell model using the Prony series^26,57^. The average behavior of the unidirectional composites is then computed as a homogeneous, linear viscoelastic, and orthotropic material. The average strain-stress behavior of the unidirectional lamina can be described using the following equation.

1 $$\left[\begin{array}{c}{\overline{\sigma }}_{11}(t;T)\\ {\overline{\sigma }}_{22}(t;T)\\ {\overline{\sigma }}_{33}(t;T)\\ {\overline{\sigma }}_{23}(t;T)\\ {\overline{\sigma }}_{31}(t;T)\\ {\overline{\sigma }}_{12}(t;T)\end{array}\right]=\left[\begin{array}{cccccc}{C}_{11}(t;T)&{C}_{12}(t;T)&{C}_{13}(t;T)&0&0&0\\ {C}_{12}(t;T)&{C}_{22}(t;T)&{C}_{23}(t;T)&0&0&0\\ {C}_{13}(t;T)&{C}_{23}(t;T)&{C}_{33}(t;T)&0&0&0\\ 0&0&0&{C}_{44}(t;T)&0&0\\ 0&0&0&0&{C}_{55}(t;T)&0\\ 0&0&0&0&0&{C}_{66}(t;T)\end{array}\right]\left[\begin{array}{c}{\overline{\varepsilon }}_{11}^{cst}\\ {\overline{\varepsilon }}_{22}^{cst}\\ {\overline{\varepsilon }}_{33}^{cst}\\ {\overline{\gamma }}_{23}^{cst}\\ {\overline{\gamma }}_{31}^{cst}\\ {\overline{\gamma }}_{12}^{cst}\end{array}\right]$$

where *t* and *T* denote stowage time and temperature, respectively. Note that the stowage temperature has a significant effect on the time-dependent behavior of a viscoelastic material. Specifically, a long-time relaxation process of a material at low temperatures is equivalent to a short-time relaxation process at high temperatures. The temperature dependence of the relaxation modulus is correlated to time through the time-temperature superposition principle, in which the relaxation times at two temperatures are related by a shift factor. [*C*(*t*; *T*)] is the stiffness matrix of the unidirectional lamina, each entry of which is essentially time- and temperature-dependent. Eq. (1) indicates that the overall stress level of each composite layer $\overline{\sigma }(t)$ is measured as a function of storage time and temperature when the lamina is subjected to a constant deformation ${\overline{\varepsilon }}^{cst}$.

The second homogenization step utilizes the obtained unidirectional composite (yarn) properties to analyze the behavior of the woven composite by constructing a mesoscale RVE, which homogenizes the yarn and matrix properties in the mesostructure of the woven composite and yields the relaxation ABD matrix as a result. The ABD matrix represents the overall stiffness of the laminate, which is used to relate the deformation strains and curvatures of the middle surface of a laminate to the resultant forces and moments exerted on the laminate.

2 $$\left(\begin{array}{c}{N}_{x}(t;T)\\ {N}_{y}(t;T)\\ {N}_{xy}(t;T)\\ {M}_{x}(t;T)\\ {M}_{y}(t;T)\\ {M}_{xy}(t;T)\end{array}\right)=\left(\begin{array}{cccccc}{A}_{11}(t;T)&{A}_{12}(t;T)&{A}_{16}(t;T)&{B}_{11}(t;T)&{B}_{12}(t;T)&{B}_{16}(t;T)\\ {A}_{12}(t;T)&{A}_{22}(t;T)&{A}_{26}(t;T)&{B}_{12}(t;T)&{B}_{22}(t;T)&{B}_{26}(t;T)\\ {A}_{16}(t;T)&{A}_{26}(t;T)&{A}_{66}(t;T)&{B}_{16}(t;T)&{B}_{26}(t;T)&{B}_{66}(t;T)\\ {B}_{11}(t;T)&{B}_{12}(t;T)&{B}_{16}(t;T)&{D}_{11}(t;T)&{D}_{12}(t;T)&{D}_{16}(t;T)\\ {B}_{12}(t;T)&{B}_{22}(t;T)&{B}_{26}(t;T)&{D}_{12}(t;T)&{D}_{22}(t;T)&{D}_{26}(t;T)\\ {B}_{16}(t;T)&{B}_{26}(t;T)&{B}_{66}(t;T)&{D}_{16}(t;T)&{D}_{26}(t;T)&{D}_{66}(t;T)\end{array}\right)\cdot \left(\begin{array}{c}{\varepsilon }_{x}^{cst}\\ {\varepsilon }_{y}^{cst}\\ {\gamma }_{xy}^{cst}\\ {\kappa }_{x}^{cst}\\ {\kappa }_{y}^{cst}\\ {\kappa }_{xy}^{cst}\end{array}\right)$$

where the sub-matrices [*A*] and [*D*] represent the extensional and bending stiffness properties of the laminate, respectively, and [*B*] represents the coupling between the in-plane and out-of-plane loads and deformations. The ABD matrix is not constant but instead varies with time and temperature for viscoelastic composite laminates. This means that When a composite laminated structure is subjected to constant deformation characterized by applied strains (${\varepsilon }_{x}^{cst}$, ${\varepsilon }_{y}^{cst}$, ${\gamma }_{xy}^{cst}$) and curvatures (${\kappa }_{{{{{{{{\boldsymbol{x}}}}}}}}}^{cst}$, ${\kappa }_{{{{{{{{\boldsymbol{y}}}}}}}}}^{cst}$, ${\kappa }_{{{{{{{{\boldsymbol{xy}}}}}}}}}^{cst}$) at a given temperature *T* and for a specific stowage period *t*, the resulting forces and moments exerted on the structure will depend on the stowage time and temperature.

The core of the multiscale modeling approach is indeed to calculate the stowage time- and temperature-dependent stiffness matrices, such as the lamina stiffness matrix [*C*(*t*; *T*)] and the ABD matrix for laminate stiffness. This can be achieved by implementing the homogenization approach within the standard finite element method (FEM). For the calculation of the lamina relaxation stiffness [*C*(*t*; *T*)], a microscale RVE is created with periodic boundary conditions, and six in-plane specific loading conditions, including three axial loadings and three shear loadings, should be performed subsequently. Differently, the ABD matrix calculation requires the implementation of periodic boundary conditions that enforce midplane strains and out-of-plane curvatures of the mesoscale RVE as a homogenized thin Kirchhoff plate. It aims to capture the overall deformation of the woven composite when it is subjected to not only in-plane extensional loads but also out-of-plane bending moments. In summary, the calculation results of the micro-RVE analysis are the time- and temperature-dependent stiffness matrix [*C*(*t*; *T*)] of the unidirectional composite (yarn), while the result yielded by the meso-RVE analysis is the time- and temperature-dependent ABD matrix for the composite laminates. Finally, the ABD matrix can be used to define the constitutive behavior of composite laminates in the standard finite element analysis, facilitating analysis of the viscoelastic behavior of any thin-walled composite laminates. It should be noted that, for unidirectional laminates, the two-step homogenization procedure can be simplified by avoiding the calculation of the ABD matrix, since the laminate code of unidirectional laminates can be defined via built-in functions in commercial FEM packages.

### Viscoelastic behavior of DCBs

Following the above-mentioned modeling strategy, many studies have been reported to understand the viscoelastic behavior of composite deployable structures during long-term stowage. A summary of these studies is given in Table 2. Long et al.^58^ derived and implemented two solution techniques, i.e., quasi-elastic and direct integration, into an anisotropic viscoelastic shell formulation, and captured nonuniform deformation in the bending of thin-ply HSCs. Hamillage et al.^59^ investigated the bending relaxation behavior of thin-ply composites and verified the theoretical model predictions with experiments by performing column bending tests. Kwok et al.^53^ analyzed the deployment performance of plain-weave composite tape-spring shells that are deployed after being held folded for a given period of time. Zhang et al.^60^ analyzed the bistable behavior of C-shaped composite shells with viscoelastic material properties, and reported that the principal curvature of the shell’s second stable state increases as the applied temperature and relaxation time increase. Brinkmeyer et al.^61^ studied the stowage effects on the deployment behavior of storable bistable tape springs, and found that the deployment time increases predictably with stowage time and temperature, and for cases where stress relaxation is excessive, the structure is shown to lose its ability to deploy autonomously. Fernandes et al.^62^ proposed a numerical approach that simulates the viscoelastic relaxation of composite tape-spring hinges. An et al.^63^ developed a user-friendly RVE analysis plug-in tool in Abaqus/CAE to rapidly estimate the effective orthotropic viscoelastic properties of unidirectional composites by taking as input the microstructure geometry as well as the known properties of fibers and matrix. The tool was utilized to simulate the influence of modulus relaxation on the deployment dynamics of a composite tape-spring hinge. Gomez-Delrio and Kwok derived an analytical solution for the recovery of a composite plate after stowage and studied the stowage and recovery of a deployable lenticular boom^64^. Deng et al.^65^ simulated the viscoelastic strain energy relaxation in the long-term stowage periods of CTLTs made of unidirectional laminates. Guo et al.^66^ built a gravity-unloading system and performed dynamic deployment tests on the deployable booms before and after stowing them for 6 and 10.5 months. The experimental results provide verification for the multiscale simulation methods and demonstrate that the deployment time increases with the increase in stowage time. Most of the existing studies have analyzed the viscoelastic behavior of HSC deployable structures, however, there is a gap in exploring the viscoplastic deformation of these structures. The viscoplastic deformation involves the permanent deformation of the composite materials under the influence of both time-dependent and plastic deformation mechanisms.

**Table 2:**
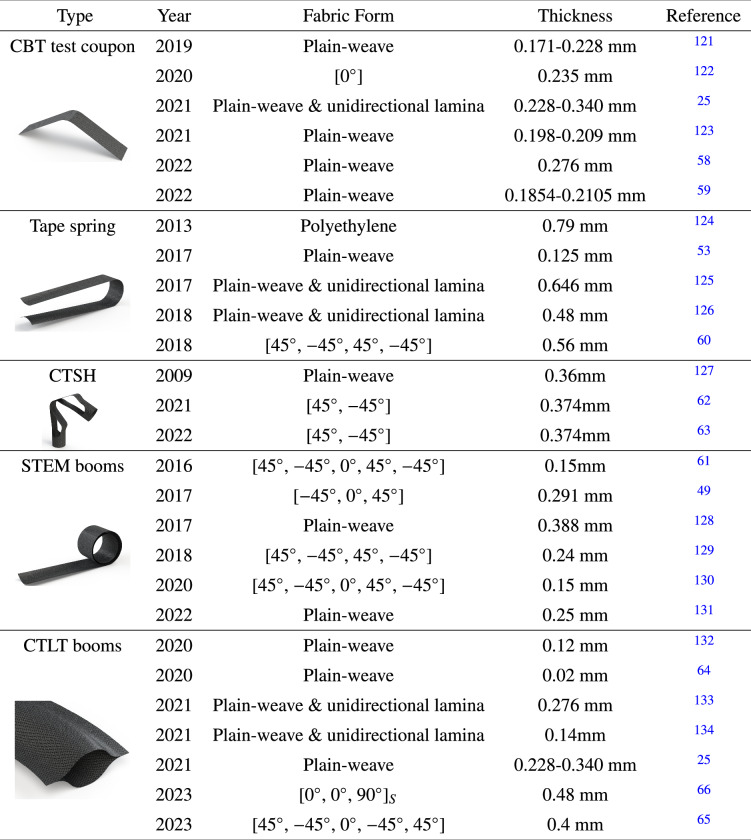
Summary of studies on stowage effects for DCBs

### Effect of boom-hub interface

The composite deployable booms need to be connected to the central deployment devices such as the hub, and the boom-hub interface provides the root boundary conditions of the boom. There are three main fixation conditions^67^: (1) The root cross section of the boom is fully fixed. This method requires a long length from the fixed end to flatten and wrap the boom to avoid damage. (2) The root cross section of the boom is partially fixed. In this case, the length needed for cross section shape transition is smaller but this method also lowers the bending stiffness of boom in the fully deployed condition. (3) The root cross section of the boom is totally flattened so that the boom can be wrapped around the hub in a very compact manner. But this method weakens the load-carrying capacity of boom significantly. So the interface between the boom and the hub must be comprehensively investigated and designed to achieve efficient storage and avoid failure.

Okuizumi^67^ designed a novel metallic spring root hinge as the interface of composite boom. The root hinge is inserted between boom and hub, the end connected to the hub is fully fixed while other end connected to the boom can be collapsed with boom. This concept can improve storage efficiency and ensure the stiffness of boom after deployment. Pellegrino^68^ proposed a new interface for CTLT booms. By removing material from the fix end of CTLT boom to achieve the small radius folding and avoid curvature localization which will cause the failure of composites.

Mallikarachchi^69^ applied four different boundary conditions in the numerical simulations of the dynamic deployment of CTSH to investigate the sensitivity of the response to the root boundary conditions. The result shows that the boundary condition significantly affect the results of the simulations, which also illustrates the necessity of experiment to assist the appropriate selection of boundary conditions in simulation. But the on-ground test is still challenged by the design of clamp conditions and gravity compensation system and the test results also affected by the friction, air resistance and other factors.

### Time-dependent failure and optimization

The investigation of the failure mechanisms of HSC deployable structures is essential to ensure that the structure’s operational performance is as intended. In order to predict the failure behavior of composite laminates, several experimentally based failure criteria have been developed. Mallikarachchi et al.^7^ proposed a method to identify potential damage areas by searching for the largest midplane strains and comparing these peaks with material damage values to estimate the safety of the structure. Mallikarachchi and Pellegrino^27^ presented a failure criterion suitable for two-ply carbon fiber laminates considering three different loading cases: failure due to in-plane, bending, and combined in-plane and bending loads. The failure behavior predicted by this criterion is in high agreement with experiments and this criterion has been successfully applied to topology and shape optimization problem of composite deployment structures^28,30^.

It’s important to note that when dealing with long-term stowage of H materials, not only does the stiffness of the material degrade, but the strength of the composite laminates can also be influenced^70^. Ubamanyu et al.^71,72^ proposed a Flattening to Rupture test to effectively load composite coupons under long-term bending, enabling the measurement of time-dependent rupture and identification of the underlying time-dependent failure mechanisms. Numerical simulation methods were also developed to understand the sequence of rupture events and the parameters that affect the time-dependent rupture. Furthermore, the accumulation of plastic deformation in laminated composite structures during long-term stowage is indeed a significant challenge when it comes to modeling and predicting the behavior of these materials. Plastic deformation, which is permanent and non-recoverable deformation in the material, can occur in composite laminates for various reasons, and it can have a critical impact on the structural integrity of these components over time. Zhang et al.^73^ developed a nonlinear viscoelastic-viscoplastic constitutive model for epoxy polymers. Matsuda et al.^74^ analyzed in-plane elastic-viscoplastic deformation of carbon fiber/epoxy laminates using a homogenization theory of nonlinear time-dependent composites. Megnis et al.^75,76^ useds Schapery’s nonlinear viscoelastic, viscoplastic material model to characterize the inelastic response of glass fiber epoxy composites. In addition, in the context of optimization design of composite depolyable structures, most of existing optimization problems were constructed without considering the material degration during the stowage effects^29,30,35,77^. The long-term stowage effects of the HSC deployable structures should be accounted for in the future work to ensure the recovery accuracy of space mechanisms such as antennas.

## Manufacturing of composite deployable structures

Resin, as the matrix material in composite materials, exhibits various characteristics during its curing process, such as thermal expansion coefficient, curing shrinkage, and mechanical properties, which directly affect the generation and distribution of residual thermal stress in the composite material. These residual stresses can lead to unpredictable shape changes and performance degradation, or even material failure during application. Therefore, it has become an urgent and important issue to enhance the mechanical performance and shape recovery precision of composite deployable structures by considering resin properties, thermal stress control, and manufacturing processes.

### Enhancing resin performance and controlling thermal stress

Deployable composite structures for space applications have specific resin performance requirements. These requirements aim to ensure that composite deployable structures can meet the unique demands and operating conditions in space. The resin performance requirements can be summarized as follows:

1. Mechanical properties: The resin should possess sufficient strength, stiffness to withstand stresses and loads in the space environment. Additionally, the resin should exhibit good fatigue and impact resistance to handle loading conditions during long-term usage.
2. Thermal properties: Extreme temperature variations exist in the space environment, ranging between −80 °C and 100 °C. Therefore, the resin needs to exhibit excellent thermal stability and high-temperature resistance to maintain structural stability and strength.
3. Viscoelastic characteristics: Viscoelastic materials exhibit deformation and stress relaxation, also negatively impact the deployable behavior and shape recovery accuracy of composite deployable structures. Consequently, viscoelasticity should require appropriate correction and control.
4. Dimensional stability: Composite deployable structures for space applications need to possess excellent dimensional stability. The resin should have a low coefficient of thermal expansion and excellent linear thermal shrinkage performance to ensure stability and precision of the structure during temperature variations.
5. Radiation resistance: Radiation, such as cosmic rays and ultraviolet radiation, exists in the space environment. The resin needs to possess appropriate radiation resistance to prevent damage to the structure and resin performance.
6. Processability: The processability of the resin are crucial for manufacturing composite deployable structures for space applications. The resin should exhibit good flowability and moldability to facilitate the fabrication and assembly of composite materials, and consider the curing characteristics and controllability of the resin comprehensively.

In conclusion, the resin performance requirements for composite deployable structures are stringent. Therefore, it is necessary to comprehensively consider these requirements during resin design and selection, and conduct thorough evaluation and testing to ensure that the resin can meet the specific demands of space applications and guarantee the reliability and safety of composite deployable structures.

In additionally, residual thermal stresses and deformations during the processing and shaping of composite structures are not only intricately connected to the performance of the resin, but also are generated by mismatches of thermal expansion coefficients of component materials, material anisotropy, structural forms, lay-up methods, chemical shrinkage, mismatched coefficients of thermal expansion between molds and components, mold-component interfaces, allowance for defects (such as voids and inclusions), and curing processes. Many researchers have extensively studied these issues using experiments, analytical methods, and numerical simulations.

Experimental testing is the most direct and accurate method for studying the residual thermal stresses and deformations generated during the curing process of composite laminates. Bogetti and Gillepie^78^ investigated the curing deformation mechanism of composite laminates through experiments and found that temperature, degree of cure, and resin distribution in the thickness direction have significant influence on residual thermal stresses and deformations. White and Hahn^79^ found that reducing the curing temperature while increasing the curing time, or conducting the cooling process at a lower rate, can effectively reduce the post-curing thermal deformation. Daniel and Liber^80^ used the measured strains to calculate the residual thermal stresses in the laminates and further investigated the influence of lay-up schemes on residual thermal stresses, validating that residual stresses in the laminates can lead to transverse cracking in individual layers. Additionally, low-temperature curing and staged curing have also been shown to effectively reduce post-curing residual thermal stresses and thermal deformations^81,82^. However, experimental testing has limitations such as high costs, lengthy processes, and dependence on experimental conditions. Numerical simulations based on finite element methods can effectively address these limitations. Bogetti et al.^83^ studied the curing process of laminates with arbitrary cross-sectional shapes and boundary conditions using a two-dimensional finite element model. Satish et al.^84^ introduced shear layers in their finite element model to simulate the effect of molds on composite materials, enabling accurate prediction of thermal deformation resulting from mold-material interactions. Some studies have investigated the factors affecting the curing deformation in asymmetric laminates. The results showed that thermal deformation in asymmetric laminates is closely related to the dimensions of the laminates, and when the length and width increase to a certain extent, the stable shape after curing changes from saddle-shaped to cylindrical shell-shaped. The curing deformations increase with an increase in curing temperature, while the cooling rate has little effect on thermal deformation^85–87^. Compared to experimental testing and numerical models, theoretical modeling offers lower computational complexity and faster solution speeds, allowing preliminary results to be obtained in a shorter period of time. Hahn and Pagano^88^ proposed an analytical model based on classical laminate theory for estimating residual thermal stresses in laminates. Xiong et al.^89^ developed a micromechanical theoretical model to predict the residual thermal stresses in plain-weave composite laminates and provided an analytical expression for estimating thermal stresses.

Summarily, there are numerous factors that influence thermal deformation and thermal stress, including component material properties, material anisotropy, structural form, lay-up scheme, interaction between molds and components, curing process and parameters, chemical shrinkage, etc.^90,91^. During the preparation of deployable composite structures, residual stress is mainly caused by internal and external stress sources. Among them, the main internal stress source is the shrinkage of the resin during curing and the mismatch of thermal expansion coefficients, while factors such as specimen shape, specimen-mold interactions, and process conditions constitute the external stress sources. Due to the limitations of material inherent properties, it is impossible to completely eliminate the existence of residual stress. Therefore, the key lies in finding effective ways to minimize or mitigate the impact of residual stress during the preparation process. Specific methods include: 1) optimizing the curing process (such as adjusting curing time, temperature, and curing degree gradient); 2) introducing expandable materials; 3) improving interface properties; 4) using new curing technologies such as electron beam curing, UV curing, and laser curing; 5) applying external prestress. These measures belong to external intervention methods, which theoretically can reduce the impact of internal residual stress to a certain extent, but still face many technical challenges in practical operation. Among them, optimizing the curing process is a relatively effective method, but it is limited by various factors, so the effect is not significant. Due to the dispersion, anisotropy, and resin characteristics of composite materials, it is extremely difficult to find monomers that are compatible with them, leading to difficulties in implementing the method of filling foreign materials. In addition, the interface properties of composite materials involve the microscale and are difficult to intervene from a macro perspective. New curing technologies require extremely high requirements for process, material compatibility, and equipment precision, often requiring high cost investment. Recent research has shown that applying prestress may effectively reduce residual thermal stress^92^, but this method is still in its infancy and its mechanism is not yet clear. In theory, applying prestress can not only induce multistable characteristics of deployable composite structures, but also reduce residual stress in composite materials. However, how to find a balance point in deployable composite structures that does not interfere with each other still needs further research. This process involves multi-disciplinary fields such as multi-physical field theory, composite mechanics, and material forming processes, and still requires a large amount of theoretical, numerical, and experimental research support to find effective solutions for controlling thermal deformation and thermal stress.

### Fabrication process

Compared to other composite deployable structures, the lenticular composite deployable boom poses greater complexity and challenges in the manufacturing process due to the unique structural form. The preparation process is a crucial factor in achieving the folding and deploying functions and controlling the shape accuracy. The lenticular composite deployable boom, being thin-walled structures, require considerations from various aspects such as process cost, feasibility, and quality precision. Currently, vacuum bag or autoclave methods are widely used for manufacturing lenticular composite deployable boom^12,13,93–99^. In this section, detailed introductions of the manufacturing process by Bai and his colleagues^78–82^ are provided as follows:

1. The mold, prepreg, and thermoplastic film for molding were prepared, as shown in Fig. 4a.

**Fig. 4:**
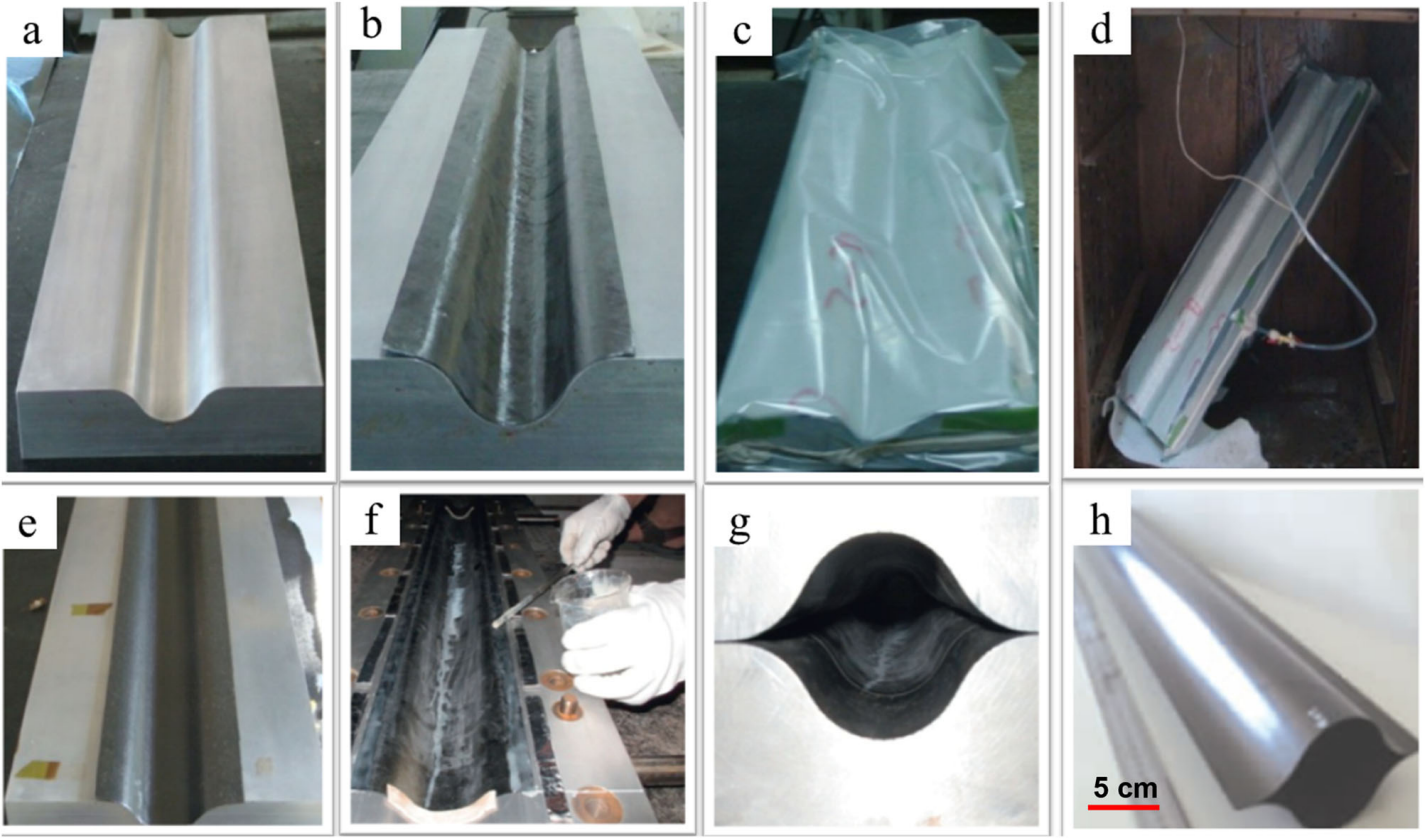
Vacuum bag and co-bonding processes of thin-walled lenticular deployable composite boom (DCB)^13^. **a** Mold. **b** Stacking prepreg. **c** Bagging. **d** Curing. **e** Semi-shell specimen. **f** Coating adhesives. **g** Clamping of both molds. **h** DCB [Adapted from ref. ^13^ with permission].
2. The prepreg was cut and laid out according to the scheme. Air trapped between the layers was minimized during the laying process, as shown in Fig. 4b.
3. Once the prepreg was laid, auxiliary materials were positioned on top of it, followed by the encasement of the vacuum bag using a sealing strip, as depicted in Fig. 4c.
4. The vacuum-bagged mold was placed into a baking box. During the cooling process, the pressure should be maintained until the temperature of the part was lower than 60 °C, as shown in Fig. 4d.
5. After full curing, one half of lenticular composite deployable boom from was removed from the mold, as shown in Fig. 4e. The bonding edges of one half of lenticular composite deployable boom were trimmed and polished. Subsequently, adhesive was applied to one of the half molds, and then the two halves were pressed together, lastly, the lenticular composite deployable boom specimen was obtained, as shown in Fig. 4f–h.

**Fig. 5:**
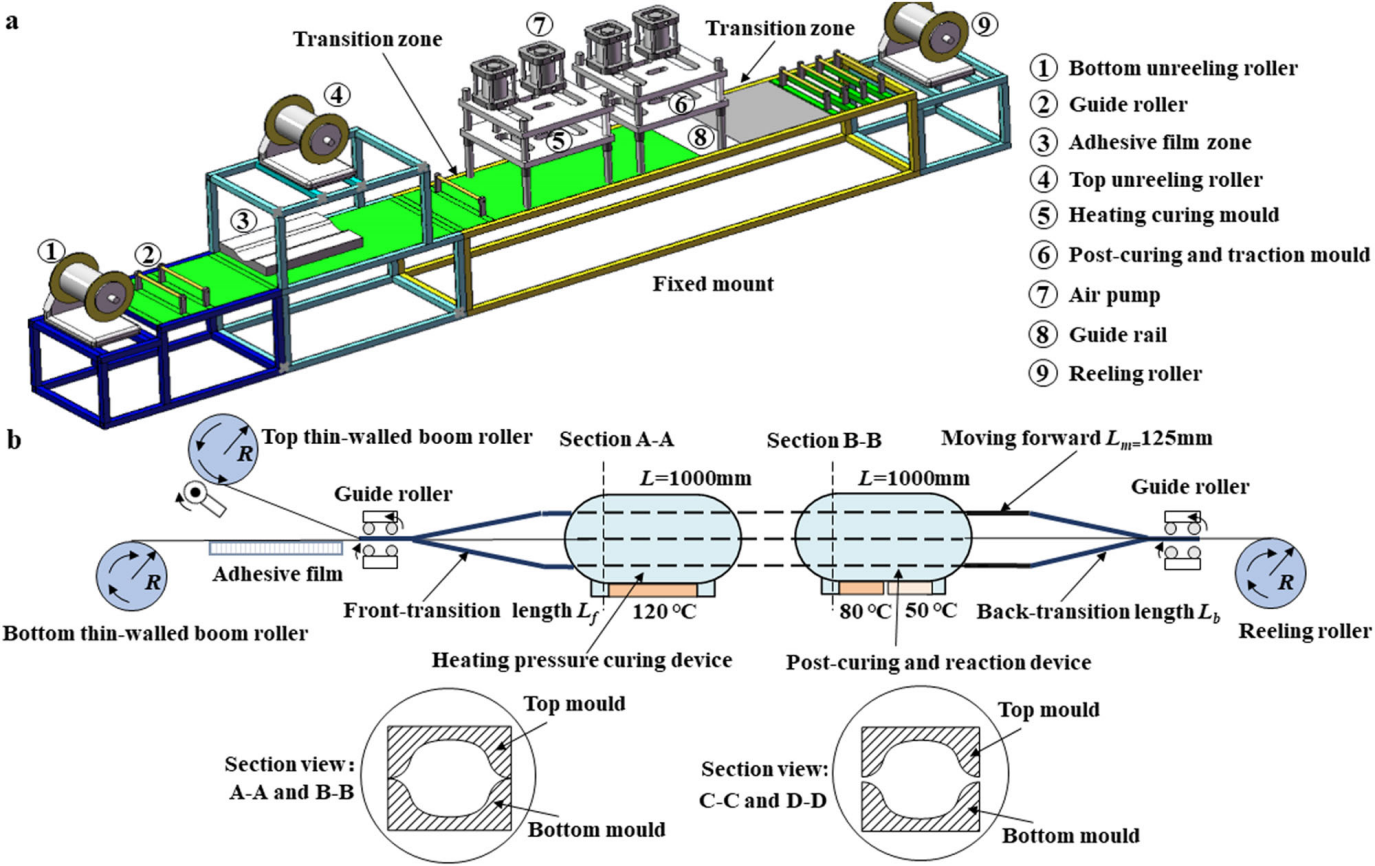
Continuous forming process of ultra-long thin-walled lenticular Deployable Composite Boom (DCB)^100^. **a** Continuous curing molding equipment of semi-shell. **b** Continuous adhesion process design.

The vacuum bag method offers advantages like cost-effectiveness, feasibility, high-precision, and broad applicability, making it a practical approach for producing composite deployable structures. However, it faces challenges when fabricating ultra-long booms (≥20 m) due to constraints related to space, molds, and equipment. Advanced pultrusion (ADP) technique enables automated production of composite deployable structures with unlimited continuous length, providing another technical means for manufacturing ultra-long lenticular composite deployable boom. The specific manufacturing process involves using prepreg material to produce composite deployable structures with specific cross-sectional shapes through steps such as preforming, hot pressing, post-curing, and cutting. Based on the ADP technique, Zhang et al.^100^ fabricated a 75 m lenticular composite deployable boom using the continuous curing process, achieving continuous curing of one half of boom and continuous bonding of the boom, as shown in Fig. 5a and b.

The ADP technique offers high automation and versatility, making it suitable for ultra-long composite deployable structures. However, it may have slightly lower precision compared to the vacuum bag method. The vacuum bag method is well-suited for structures with complex cross-sectional shapes and less stringent dimensional requirements, but it demands careful selection of resin and adhesive, along with precise control over the curing process.

The manufacturing process of composite deployable structures has the complexity of multi-physical field coupling, in which the interaction between fibers and matrix has an important impact on the quality and performance of the finished product. This mainly involves the resin flow, crystallization, compaction process, and the properties of the interface layer. During the curing process, as the temperature of the outer surface of the component changes, the internal resin undergoes volume shrinkage under the combined effect of thermal effects and polymerization crosslinking reactions. In addition, there is a significant difference in the thermal expansion coefficients between fibers and resins, which may cause resin to undergo extrusion or relaxation around the fibers and flow under external forces. This uneven flow may result in uneven distribution of fiber volume fractions, local resin enrichment or depletion, and weakened interfacial adhesion between fibers. The compaction process of composite materials is related to the resin infiltration flow, and its compaction behavior and extent affect the uniformity of resin flow. At the same time, the fiber skeleton and material properties also affect the compaction degree, resin viscosity, and curing effect. In summary, the curing shrinkage and thermal expansion coefficient mismatch between fibers and matrix are the inherent factors that cause thermal deformation and thermal stress in composite materials. The more severe their effects, the more prone the finished product is to surface precision issues such as porosity and wrinkling, which seriously affect its mechanical properties such as strength, stiffness, and durability. It may even lead to structural failure issues such as delamination and cracking, seriously threatening product reliability and safety^101,102^. Therefore, in-depth understanding of the interaction mechanism between fibers and matrix is key to reducing thermal deformation and thermal stress, while also providing important theoretical guidance for improving the dimensional precision, surface quality, and mechanical properties of composite products.

This section provides a review of the preparation process of deployable composite structures, deeply analyzes the internal and external factors that affect their forming quality, mechanical properties, and shape recovery precision. Through comprehensive analysis, it summarizes the current problems and challenges faced by deployable composite structures in the curing process. According to the authors’ knowledge, there is currently no other literature that comprehensively summarizes the key elements, considerations, and technical difficulties in the design process of deployable composite structures from a production and preparation perspective.

## Space applications of composite deployable structures

### Space applications

**Fig. 6:**
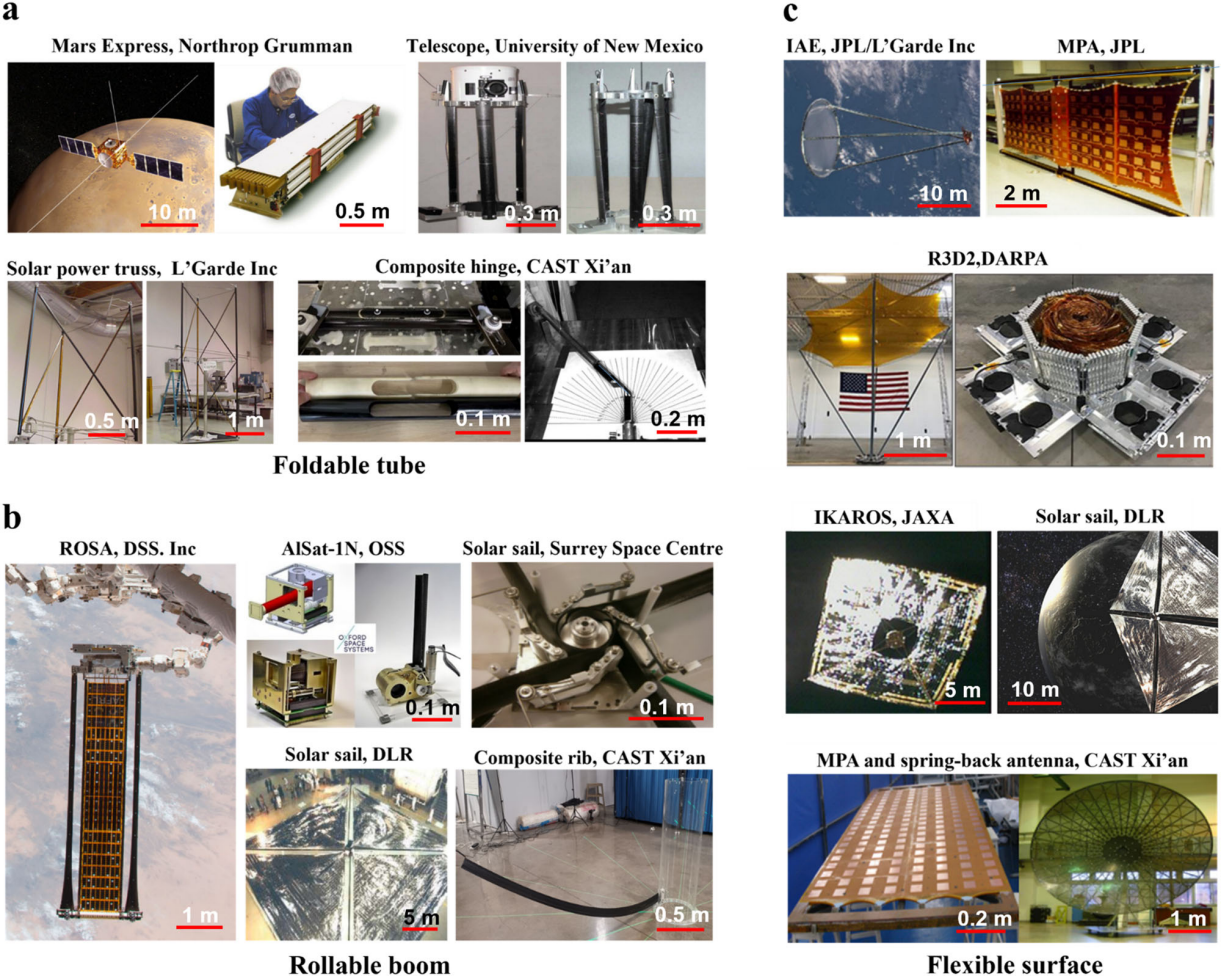
Space applications of composite deployable structures. **a** Foldable tube: Mars Express^110^, Northrop Grumman [Source: Northrop Grumman]; Telescope^144^, University of New Mexico [Reproduced from ref. ^144^ with permission]; Solar power truss^149^, L'Garde Inc [Source: L'Garde Inc]; Composite hinge^29^, China Academy of Space Technology (CAST), Xi'an. **b** Rollable boom: Roll Out Solar Array (ROSA)^137^, Deployable Space Systems (DSS) Inc [Reproduced from ref. ^137^ with permission]; Algerian Nanosatellite (AlSat-1N)^154^, Oxford Space Systems (OSS) [Source: Oxford Space Systems (OSS)]; Solar sail^20^, Surrey Space Centre [Reproduced from ref. ^20^ with permission]; Solar sail^150^, German Aerospace Center (DLR) [Reproduced from ref. ^150^ with permission]; Composite rib^66^, China Academy of Space Technology (CAST), Xi'an. **c** Flexible surface: Inflatable Antenna Experiment (IAE)^111^, Jet Propulsion Laboratory (JPL)/ L'Garde Inc [Source: National Aeronautics and Space Administration (NASA)]; Membrane Phased-array Antenna (MPA)^163^, Jet Propulsion Laboratory (JPL) [Source: Jet Propulsion Laboratory (JPL)]; Radio Frequency Risk Reduction Deployment Demonstration (R3D2)^112,113^, Defense Advanced Research Projects Agency (DARPA) [Source: Defense Advanced Research Projects Agency (DARPA)]; Interplanetary Kite-craft Accelerated by Radiation Of the Sun (IKAROS)^118^, Japan Aerospace Exploration Agency (JAXA) [Reproduced from ref. ^118^ with permission]; Solar sail^116^, German Aerospace Center (DLR) [Reproduced from ref. ^116^ with permission]; Membrane Phased-array Antenna (MPA) and spring-back antenna^119,120^, China Academy of Space Technology (CAST), Xi'an.

Figure 6 and Table 3 show the commonly used composite deployable structures and their space applications. Examples include solar arrays, antennas, booms, radar reflectors, solar sails, deployable radiators, and boom-mounted instruments. Roll Out Solar Array (ROSA) is designed to generate power for spacecraft, particularly satellites and other missions in space. It is known for its compact, lightweight, and roll-out design, which allows for efficient storage during launch and easy deployment in space^103^. Oxford Space System (OSS) has developed an X-band wrapped-rib antenna, with a 2.7 m-diameter parabolic reflector supported by 48 CFRP CTMs^104^. At the test of the primary structure, the derivation from the ideal shape has not changed after the first stowage test. Surrey Satellite Technology Ltd (SSTL) and OSS have confirmed to build and launch a wrapped-rib antenna with a 3m-diameter reflector. The antenna has successfully completed ground-based tests and is now ready to demonstrate its performance in orbit^105^. The surface accuracy of the reflector is strongly related to the bending stiffness of the ribs^106^. HSC structures can be used as not only the support frames, but also the deployable reflector. Tan et al.^107^ developed a 4.6m-diameter stiffened spring-back antenna with deployable reflector. Soykasap et al.^108^ did the optimization study of Tan’s work and proposed a 6m-diameter thin shell spring-back reflector antenna, with the reflector made of CFRP. The analyses showed that the antenna met the specifications required for Ku-band operation. HSC can also be used in foldable hinges. The European Space Agency (ESA) launched a foldable antenna, the Mars Advanced Radar for Subsurface and Ionospheric Sounding antenna in 2003^109^. The main deployable members of the antenna were three foldable tube hinges made of a Kevlar and glass fiber composite material. Because of viscoelastic behavior of the material and complex space environmental conditions, one of the hinges had not completely locked into space^110^, and the deployment was after a year of delay.

**Table 3:** Summary of space applications

Space application	Part	Fold/deployment method	Material	R&D department	Maturity
ROSA^103,135–137^	Rollable boom	Flattened and rolled up	Carbon fiber/epoxy	Deployable Space Systems (DSS) Inc	+++
Mars Express spacecraft^110,138,139^	Foldable tube	Hinge folding	Kevlar and fiberglass/epoxy	Northrop Grumman	+++
MSAT-1/2^140^	Reflective surface	Shell winding	Carbon fiber/epoxy	Hughes	+++
TacSat-2^141^	Foldable tube	Hinge folding	Elastic memory composite	Composite Technology Development Inc	+
Precision optical telescope mirror^142,143^	Foldable tube	Hinge folding	Carbon fiber/epoxy	Foster-Miller	+++
Meade LightBridge Dobson Newtonian telescope^144^	Foldable tube	Hinge folding	Carbon fiber/epoxy	University of New Mexico	+
CubeSat^28,145^	Foldable tube	Hinge folding	Carbon fiber/epoxy	Caltech	+
SAR antenna^146^	Reflective surface	Shell folding	Carbon fiber/epoxy	Caltech	+
CubeSat^147,148^	Reflective surface	Shell folding	Fiberglass/epoxy and silicone	Caltech	+
Space Solar Power Truss^149^	Foldable tube	Inflatable rigidizable	Carbon fiber/Polyurethane	L’Garde Inc	++
Solar sail^150–152^	Rollable boom	Flattened and rolled up	Carbon fiber/epoxy	DLR and ESA	++
AISat-1N^153,154^	Extendable boom	Flattened and rolled up	Carbon fiber/epoxy	OSS	+++
CubeSat^155,156^	Lattice boom	Helically wound	Carbon fiber/epoxy	University of Limerick	+
Membrane antenna^157–160^	Reflective surface	Shell folding	Carbon fiber/silicone	Technische Universitaet Muenchen	+
Solar sail^20,161^	Rollable boom	Flattened and rolled up	Carbon fiber/epoxy	Surrey Space Centre	++
Solar sail^162^	Rollable boom	Flattened and rolled up	Carbon fiber/epoxy	Air Force Research Laboratory (AFRL)	++
Wrapped-rib antenna^104,105^	Rollable rib	Flattened and rolled up	Carbon fiber/epoxy	OSS	++
IN-STEP inflatable antenna experiment (IAE)^111^	Reflective surface/limbs	Inflatable deployment	Polyester membrane surface/ neoprene-coated Kevlar limbs	Jet Propulsion Laboratory (JPL) and L’Garde Inc	+++
Membrane Reflective-array^112,113^	Membrane	Flexible folding and deployment	Tissue-thin Kapton	DARPA	+++
Membrane phased-array antenna^163^	Thin-film surface/fram	Flexible folding and deployment	Polyimide/lightweight graphite composite	Jet Propulsion Laboratory (JPL)	+++
Deployable membrane antenna/ Collapsible Tube Masts^114–116^	Reflective surface/boom	Collapsible and deployment	Polyimide	ESA and DLR	++
IKROS solar sail^117,118^	Sail surface	Flexible folding and deployment	Polyimide	JAXA	+++
Pujiang 2- membrane antenna	Reflective surface/boom	Coiling stows and deployment	Polyimide/CFRP	Shanghai Institute of Aerospace System Engineering	+++
Membrane antenna *&* Spring-back antenna^119,120^	Reflective surface/boom	Coiling stows and deployment	Polyimide/CFRP	CAST, Xi’an	++

+++: In orbit validated, ++: Comprehensively ground-tested, +: Prototype development.

Flexible membrane deployable antennas are mainly in the form of inflatable parabolic type, electrostatically molded deployable antennas, and supple film tensioned antennas. The inflatable and flexible tensioning types are the two types with more research. The development of inflatable antenna has gone through a long and tortuous development path, and IN-STEP inflatable antenna experiment (IAE)^111^ in 1996 undoubtedly pushed the inflatable space antenna technology to a milestone new peak. The inflatable antenna in the test mainly consists of an inflatable reflector combination device and a ring/limb support structure, and the reflector comprises 62 aluminum-plated, triangular polyester film diaphragms about 7m thick. The ring/limb structure is made of neoprene-coated Kelvar, and the ring supports the edge of the reflector assembly. Flexible tensioned membrane deployable antenna refers to a new type of sizeable space antenna that integrates and innovates flexible electronics, thin-film material, flexible structure, and flexible unfolding technology, characterized by lightweight, high spreading ratio, high gain, and beam flexibility. The reflective surface of a membrane deployable antenna usually consists of a thin-film composite material, such as polyimide, which can realize the advantages of a low-density antenna structure and a high spreading ratio. Flexible tensioned membrane deployable antennas come in two forms: reflective-array antennas and direct-radiation array antennas. A reflector array flexible membrane antenna is a passive antenna; the light film structure forms the reflecting surface, and the feed is placed outside the reflecting surface. A typical space application is Radio Frequency Risk Reduction Deployment Demonstration (R3D2) supported by Defense Advanced Research Projects Agency (DARPA)^112,113^. The phased-array antenna is active; the reflecting surface is formed by a deployable thin-film structure, and the Transmitter and Receiver(T/R) module is integrated into the membrane structure to realize the function of the antenna. Typical space applications are, for example, the 40 m^2^ deployable synthetic aperture radar (SAR) antenna and solar sails designed by ESA and German Aerospace Center (DLR)^114–116^. The composite material was used in the membrane’s lightweight design, and a CFRP boom was developed. Japan Aerospace Exploration Agency (JAXA) researched the IKROS composite solar sails and validated them in orbit in the 2010s^117,118^. Also, the Shanghai Institute of Aerospace System Engineering achieved the Pujiang 2-membrane composite antenna flighted test, and the China Academy of Space Technology (CAST) designed membrane antennae and spring-back antennae, achieving the ground test^119,120^.

### Recent advances in space antenna in China

Specially, the application of composite materials in space-borne antennas is worth introducing in detail. The space-borne antenna is the space vehicle’s ”eyes” and ”ear”, used to receive the radio frequency signal of the launched satellite. It is one of the most critical core products of the spacecraft. Due to the space environment being exceptionally harsh, the antenna must overcome the carrier launch’s vibration loads, space high and low temperatures (−200 °C–150 °C), space zero gravity, and other environments. The development of antenna technology in antenna material also put forward higher requirements.

Composites can realize the comprehensive and high-index performance requirements that are difficult to meet by nano-materials, so they become the inevitable trend of material development. Carbon fiber composites, for example, have the following merits: high specific modulus (5 times higher than steel and aluminum alloys); high specific strength (3 times higher than steel and aluminum alloys); lightweight (density is half of aluminum alloy, one fifth of steel); small coefficient of thermal rise (up to 10^−6^ or less); high-temperature performance and stability; excellent corrosion resistance and radiation performance. Composites are widely used in mesh and solid surface antenna.

### Space mesh antenna

Composites, characterized by light weight, good thermal stability and high specific stiffness, are widely used in mesh antennas, accounting for about 70% or more of the number of mesh antenna parts.

**Fig. 7:**
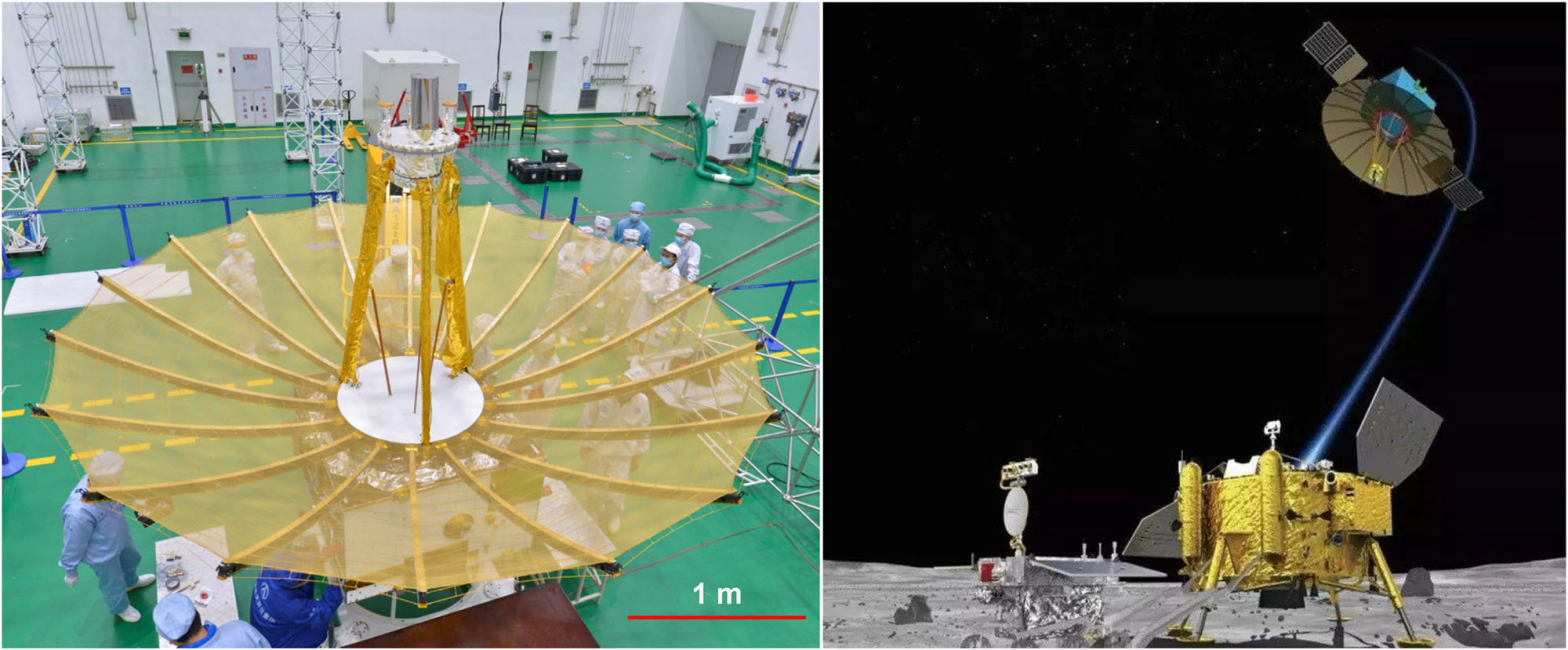
Chang’e-4’s umbrella-shaped antenna, China Academy of Space Technology (CAST), Xi’an. Space large-scale umbrella mesh antenna, applied to Chang'e-4 (China’s Lunar Exploration Project, CLEP) relay satellite, is becoming currently the farthest mesh antenna flying in the international community.

The umbrella antenna of Chang’e-4 (China’s Lunar Exploration Project, CLEP) relay satellite is currently the farthest mesh antenna flying in the international community, and the antenna works stably in the deep space environment. Chang’e-4’s antenna is shown in Fig. 7. Composites are used in design of antenna’s base. The carbon fiber base’s stiffness and thermal deformation must meet the following requirements: 1) Stiffness: axial: ≥20N μm^−1^; radial: ≥50N μm^−1^; 2) Thermal deformation: axial thermal deformation ≤70 μm; radial thermal deformation ≤60 μm.

The wrapped-rib antenna has the advantages of a high stowage ratio, simple structure, lightweight, etc. Usually, it is necessary to wrap the flexible antenna rib to the center hub to realize the stowage and then realize the self-expansion to obtain the parabolic profile by releasing the deformation energy after launching into the orbit. With the development of composite materials, a CFRP composite structure is applied to the flexible rib design of the wrapped-rib antenna. CFRP has the advantages of high specific strength, specific rigidity, creep resistance, low coefficient of thermal expansion, low thermal conductivity, high specific heat capacity, resistance to thermal shock, thermal abrasion. Space structures, made of the composites, have lighter mass, higher modulus and easier forming process.

For the development of high-throughput satellites, high-precision Ka-band umbrella antennas are more competitive. The composite rib structure is adopted to achieve the goal of a lightweight antenna. At the meantime, membrane composites are specific for developing new lightweight, high-precision umbrella antennas with preformed, stress-free, and highly stable properties.

### Space solid surface antenna

High-precision solid surface reflectors require high pointing accuracy, structural shape, and positional stability with good stability in this harsh space environment. Choosing suitable antenna reflector surface material and processing technology is necessary to solve the ultra-high profile accuracy problem. Carbon fiber composites are used for reflective surfaces and backbone on solid surface antennas. Carbon fiber composites have the merits of being lightweight, having near-zero expansion, high specific stiffness, quasi-isotropic, insensitive to moisture, and good processing performance. The solid surface reflectors of carbon fiber composites can achieve surface accuracy of up to 10 μm. Commonly used composite structural forms include honeycomb sandwich structures, laminated structures, etc.

With the development of high/ultra-high flux satellites, the antenna operating band crosses from C and KU to Ka and even Q/V bands, sub-millimeter waves, and terahertz bands. For high-precision composite solid surface antennas, such as all-composite grid structure terahertz antenna. Using a sub-block splicing-paved technique can improve the accuracy of the paving angle. On the other hand, it can effectively balance the thermal residual stress in the reflector surface and ensure the reflector surface’s overall uniformity and the profiles’ precision. At the same time, the integrated molding design of the reflector and reinforcing backbone structure is adopted, which solves the problems of mismatch of the reflector’s thermal expansion coefficient as an integration, poor thermal stability, and difficulty in maintaining the precision of the profile caused by the traditional adhesive method.

## Conclusion and outlook

This article highlighted several critical factors, including composite configuration, viscoelastic modeling, residual thermal stress, etc., that affect the shape recovery accuracy of HSC used in deployable structures in design, modeling, and manufacturing. The next step in development for HSC for space deployable structures can be concluded as follows.

### Composites design

Communications, Earth observation, data relay satellites, deep space probes, and space microwave wireless energy transmission applications have put forward ultra-large or even extensive antenna structure size requirements, the size of which can be up to 100-meter class. For larger apertures and higher profile accuracy, the requirements for composites are high specific stiffness, zero expansion coefficient, and process consistency. The field of DCBs has gained significant advancements and promising applications in recent years. This paper provides a comprehensive summary and commentary on the state-of-the-art in geometry design and composite material design for DCBs. The cut-outs design method and cross-sectional shape design method are introduced to effectively reduce folding stress levels while enhancing structural stiffness, thereby presenting new technological challenges. Configuration design methods and design criteria have rarely been studied in detail. Subsequently, it is necessary to establish the performance characterization system and failure models of the DCBs. The design theories on the composite material and structures need to be systematically carried out. Then the optimal design of the DCBs will be carried out, and the optimal design scheme can be obtained finally. With regards to wrapped-rib antenna, the ribs need to have considerable stiffness at the deployment state and be foldable without material failure during the wrapping process. With high specific strength and mature processing technique, CFRP is a kind of suitable rib material. Besides, by changing the laminate layout, the stiffness of the boom could be designed to satisfy multi-requirements, which makes it superior to isotropic material. When CFRP booms are used as the ribs of the wrapped-rib antenna, the viscoelastic behavior of the HSC would greatly affect the performance of the antenna. On the one hand, the relaxation of composite material might have an influence on the deployment process of the antenna and make the deployment time increase, even make the antenna lose the deployability. On the other hand, the surface accuracy of the reflector might be affected with the stiffness degradation and residual deformation of the ribs after repeated and long-term stowage. It should be focused on the methods to predict the viscoelastic behavior in the future work. Besides, some structural measures might be taken to weaken the viscoelastic effect and make the antenna meet the requirements. The ribs are the crucial structural members of the antenna, so it’s significant to formulate the way to design and optimize the geometric parameters and laminate layout of HSC ribs.

### Composites modeling

Developing a viscoelastic-plastic composite material model that accurately predicts the time-dependent stiffness, plastic deformation, and failure mechanisms during long-term stowage is a complex and challenging task. To address this, the following key considerations should be taken into account in future studies: (1) Comprehensive material testing and characterization are essential to comprehend the complex viscoelastic-plastic behavior of the polymer matrix. A constitutive material model is required to effectively describe the complex material behavior. (2) Multiscale modeling techniques are crucial to establish the anisotropic viscoelastic-plastic behavior of composites. This involves bridging micro and macro scales while considering the time-dependent behavior of the materials. (3) The influence of space environmental factors, such as significant temperature changes in orbit, should be considered when assessing the composite materials’ behavior during stowage. (4) Besides constitutive composite material models, there is also a lack of appropriate numerical tools tailored to address the complexities of viscoelastic-plastic behavior in composite materials. (5) Numerical optimization emerges as a valuable method for optimizing the folding and deployment performance of composite deployable structures. Constructing an optimization problem that incorporates the time-dependent behavior of the material, considerations for long-term stowage, and specific performance metrics for the composite deployable structure is essential.

### Composites manufacturing

In the design process of composite deployable structures, focus is on material selection, manufacturing processes, and functional aspects for desired functionalities and shape recovery accuracy. High-performance CFRP composites are commonly used due to unique operational conditions. Resin properties play a critical role in achieving functionality. Precise resin selection involves numerical simulation analysis and functional testing. Optimization through reinforcement or toughening is essential. Attention to residual thermal stresses and deformations during manufacturing is vital. Consideration of mold design, adhesive bonding, and surface treatment is important. Techniques like mold compensation, adhesive optimization, and laser treatment enhance bonding quality and precision. Functional validation studies viscoelastic effects on deploying behavior and shape recovery accuracy. A comprehensive review of the curing process flow, characteristics, and key elements of deployable composite structures are provided. It focuses on the resin characteristics and selection criteria in the molding process of resin-based composite materials. Additionally, it delves into the mechanisms of thermal deformation and residual stress generation during the preparation process and their impact on the final structural properties. Furthermore, a series of typical curing molding process options are also enumerated. In the outlook section, effective solutions and suggestions are proposed to enhance the comprehensive mechanical properties and shape recovery precision of deployable composite structures from the perspective of preparation processes.

## Data Availability

All data that support the conclusions are presented in the paper. All relevant data are available from the corresponding author upon reasonable request.
